# Characterisation of the murine C-type lectin receptor CLECSF8 (MCL) reveals its expression on cells of the monocyte/neutrophil lineages and an inter-dependence with Mincle, but not Dectin-2

**DOI:** 10.1186/1476-9255-12-S1-P4

**Published:** 2015-04-16

**Authors:** Bernhard Kerscher, Gillian J Wilson, Delyth M Reid, Sho Yamasaki, Janet A Willment, Gordon D Brown

**Affiliations:** 1 University of Aberdeen, Institute of Medical Sciences, Aberdeen Fungal Group, Aberdeen, UK; 2 Kyushu University, Research Center for Infectious Diseases, Fukuoka, Japan

## Background

C-type lectin-like receptors (CTLRs) play critical roles in immunity and homeostasis by recognising a great variety of microbial or endogenous ligands [1]. CLECSF8 is a member of the Dectin–2 family of CTLRs. Previous research indicates that CLECSF8 associates with the FcRγ adaptor, which is essential for surface expression and signalling through the SYK/CARD9 pathway. Recently, the mycobacterial cord factor (TDM, trehalose-6,6’-dimycolate) was identified as the ligand of CLECSF8, as shown previously for the closely related CTLR Mincle [2]. Indeed, we recently showed that CLECSF8 plays a critical role in human and murine anti-mycobacterial immunity [3]. In this study, we characterised CLECSF8 expression in the mouse under naïve and inflammatory conditions.

## Materials and methods

We used newly generated anti-CLECSF8 monoclonal antibodies (mAB) to assess receptor expression in the mouse by flow cytometry. The co-dependence of CLECSF8 and Mincle or Dectin-2 was investigated in a transfected fibroblast cell line and primary CLECSF8^-/-^ cells.

## Results

We selected mAB clone 3A4, which was able to recognise CLECSF8 by ELISA and western blot to analyse CLECSF8 expression in the mouse by flow cytometry. While CLECSF8 transcript was widely expressed, CLECSF8 protein expression was predominantly found on monocytes/macrophages and neutrophils within e. g. the peritoneal cavity, blood and bone marrow. Notably, CLECSF8 was expressed only weakly in the lung, but strongly upregulated in a pulmonary *Mycobacterium bovis* BCG infection model. *In vitro*, CLECSF8 expression on thioglycollate elicited macrophages was strongly induced upon treatment with TLR agonists or microbial stimuli.

In agreement with previous reports, our data suggests that CLECSF8 associates with the signalling adaptor FcRγ. Interestingly, surface expression of CLECSF8 in a murine fibroblast cell line was greatly enhanced by co-transfection of Mincle, but not Dectin-2. Intriguingly, Mincle expression mirrored CLECSF8 expression in our *in vitro* stimulation experiments. Further analyses on wild-type and CLECSF8^-/-^ primary macrophages *in vitro*, or cells harvested after intra-peritoneal and intra-tracheal instillation of BCG demonstrated a lack of Mincle-upregulation in the absence of CLECSF8.

## Conclusion

CLECSF8 is a predominantly monocyte/macrophage and neutrophil expressed receptor, showing significant interdependence with Mincle, but not Dectin-2.
